# Supplementary material for: Learning curve and surgical outcome of robotic assisted colorectal surgery with ERAS program
Source: Sci Rep. 2022 Nov 29;12:20566. doi: 10.1038/s41598-022-24665-w (PMC9709162; doi:10.1038/s41598-022-24665-w)
Supplement: Supplementary file 1 — Supplementary Information 1. [file 41598_2022_24665_MOESM1_ESM.zip › LSC Raw data-Ñ[▒K/120/P(6)_ñw╝╨░O▒Kñσ.pdf]

[redacted]理診斷中心 檢驗(查)報告

申請時間：2021/03/12 13:54 簽收時間：2021/03/15 16:28 檢體：Pathology
診斷醫師：吳致瑩 謝耀文 報告時間：2021/03/24 17:10 病理號：S1008380

Pathologic diagnosis:
1. Sigmoid colon, laparoscopic anterior resection --- Tubulovillous adenoma with focal high grade dysplasia. The bilateral cut ends and mesocolic lymph nodes (0/10) are free of tumor.
2. IMA root, dissection --- Unremarkable fibroadipose tissue.
3. Colon, cut end, distal, resection --- Unremarkable.

Ancillary study for diagnosis:
1. Step cut x 3 done for section E and F.
2. IHC stain for AE1/AE3 (no abnormal infiltration) and desmin (no definite disruption) done for section E.

Gross description:
The specimen consists of 1) a segment of colon, 13 cm in length and 6 cm in proximal cut end circumference, 6 cm in distal circumference, and mesocolic soft tissue. A gray tan friable polypoid tumor, 3x3x2.5 cm, is noted, 2 cm to the distal cut end. On cross section, the tumor invades to the submucosa. There are metal staples at the root of tumor. The both cut ends are free of tumor grossly. 2) IMA root, a piece of tan yellow soft tissue, 4x2x1 cm. 3) distal cut end, a piece of tan mucosa-covered soft tissue, 2.5x2x0.7 cm. Representative part for section: A) specimen 2 B) specimen 3 C) proximal cut end D) vascular cut end E-F) polyp G) mucosa near tumor root H-S) mesocolic soft tissue and lymph nodes T-U) all the residual polyp.

Note:
The diagnosis is concurred in intradepartment consensus meeting on 2021/03/24.
#T-59471_2 #M-82632_2 9 1105 00CM00
#T-C4560_2 #M-09183_2 0 1105 000000

已電子簽章

敬告：本單僅供醫師參考使用，不具任何申請效力！！
請病患妥為保管，若不慎遺失需自負個人資料外洩責任！
